# Supplementary material for: A Fungal Arrestin Protein Contributes to Cell Cycle Progression and Pathogenesis
Source: mBio. 2019 Nov 19;10(6):e02682-19. doi: 10.1128/mBio.02682-19 (PMC6867901; doi:10.1128/mBio.02682-19)
Supplement: TABLE S1 [file mBio.02682-19-st001.docx]

**TABLE S1**. Primary amino acid sequence homology between the *C. neoformans* arrestins and *S. cerevisiae* arrestins*^a^*

| ***C. neoformans* arrestin** | **BLAST program** | ***S. cerevisiae* arrestin** | **E value** | **Identity (%)** | **Query cover (%)** |
| --- | --- | --- | --- | --- | --- |
| **Ali1** | blastp | Rog3 | 1.00E-22 | 26.95 | 29 |
|  |  | Rod1 | 8.00E-13 | 23.69 | 29 |
|  | PSI-BLAST | Rog3 | 1.00E-22 | 26.95 | 29 |
|  |  | Rod1 | 8.00E-13 | 23.69 | 29 |
|  |  | Aly2 | 5.00E-07 | 25.36 | 15 |
|  |  | Art5 | 1.00E-04 | 27.14 | 7 |
|  |  | Ecm21 | 0.002 | 22.77 | 11 |
| **Ali2** | blastp | Aly1 | 7.00E-26 | 28.71 | 31 |
|  |  | Aly2 | 1.00E-11 | 41.77 | 6 |
|  |  | Csr2 | 4.00E-06 | 26.25 | 11 |
|  |  | Ecm21 | 0.025 | 25.42 | 4 |
|  |  | Spo23 | 0.31 | 25.96 | 8 |
|  | PSI-BLAST | Aly2 | 1.00E-33 | 29.11 | 30 |
|  |  | Aly1 | 7.00E-26 | 28.71 | 31 |
|  |  | Rod1 | 7.00E-09 | 29.45 | 11 |
|  |  | Rog3 | 9.00E-07 | 25.2 | 10 |
|  |  | Csr2 | 4.00E-06 | 26.25 | 11 |
|  |  | Art5 | 8.00E-04 | 26.67 | 6 |
|  |  | Ecm21 | 0.003 | 23.33 | 9 |
| **Ali3** | blastp | N/A |  |  |  |
|  | PSI-BLAST | N/A |  |  |  |
| **Ali4** | blastp | N/A |  |  |  |
|  | PSI-BLAST | N/A |  |  |  |

*^a^* The blastp and PSI-BLAST programs were used to identify amino acid sequence conservation. Alignments with an E value less than 1 were determined to be significant (N/A = not applicable).
