# Supplementary material for: A Fungal Arrestin Protein Contributes to Cell Cycle Progression and Pathogenesis
Source: mBio. 2019 Nov 19;10(6):e02682-19. doi: 10.1128/mBio.02682-19 (PMC6867901; doi:10.1128/mBio.02682-19)
Supplement: TABLE S2 [file mBio.02682-19-st002.docx]

**TABLE S2**. Primary amino acid sequence homology between the *C. neoformans* arrestins and human arrestins*^a^*

| ***C. neoformans* arrestin** | **BLAST program** | **Human arrestin** | **E value** | **Identity (%)** | **Query cover (%)** |
| --- | --- | --- | --- | --- | --- |
| **Ali1** | blastp | N/A |  |  |  |
|  | PSI-BLAST | Arrdc2 | 0.004 | 23.47 | 19 |
| **Ali2** | blastp | N/A |  |  |  |
|  | PSI-BLAST | N/A |  |  |  |
| **Ali3** | blastp | N/A |  |  |  |
|  | PSI-BLAST | N/A |  |  |  |
| **Ali4** | blastp | N/A |  |  |  |
|  | PSI-BLAST | N/A |  |  |  |

*^a^* The blastp and PSI-BLAST programs were used to identify amino acid sequence conservation. Alignments with an E value less than 1 were determined to be significant (N/A = not applicable).
