# Supplementary figures and images for: A Fungal Arrestin Protein Contributes to Cell Cycle Progression and Pathogenesis
Source: mBio. 2019 Nov 19;10(6):e02682-19. doi: 10.1128/mBio.02682-19 (PMC6867901; doi:10.1128/mBio.02682-19)

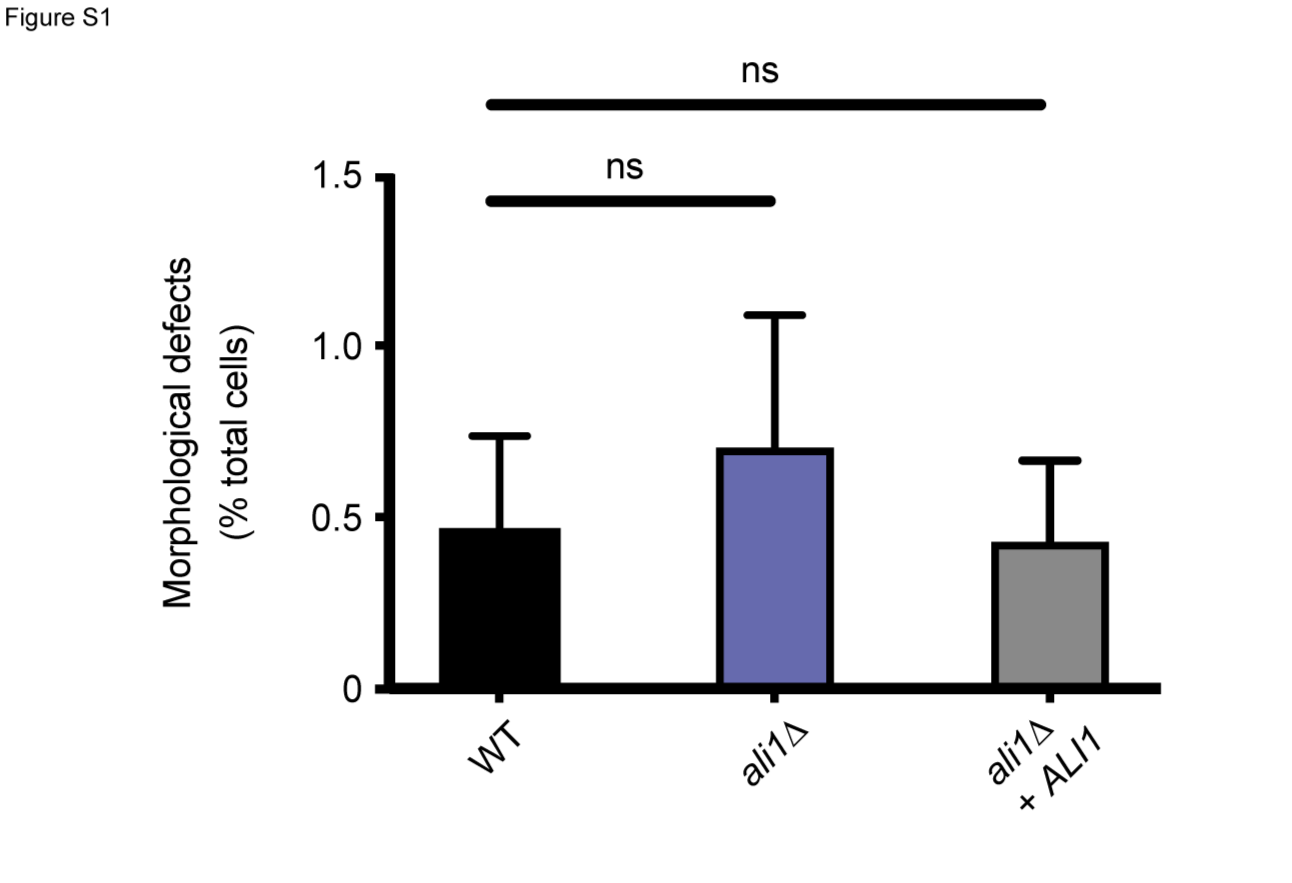

Supplement: FIG S1 [file mBio.02682-19-sf001.tif]

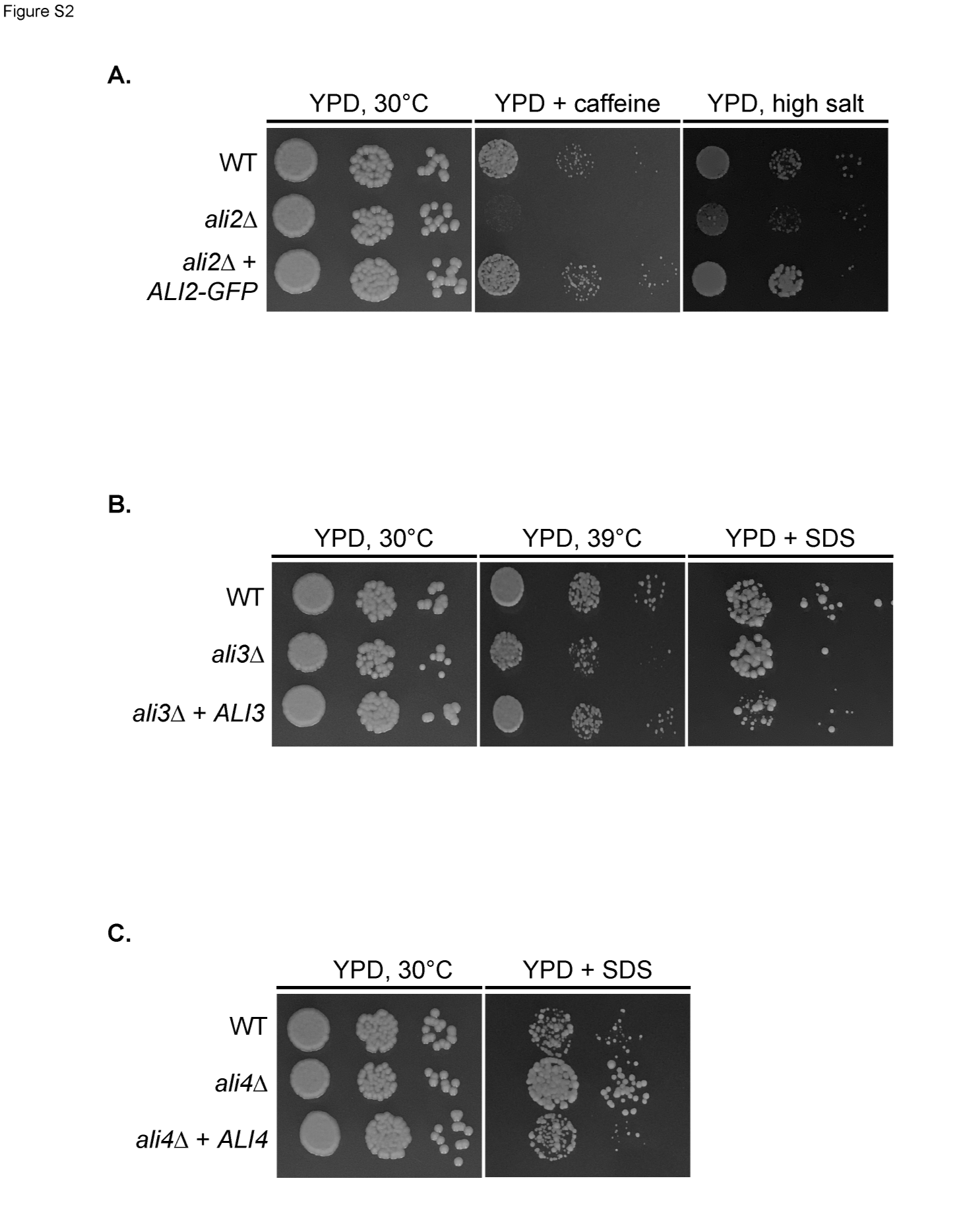

Supplement: FIG S2 [file mBio.02682-19-sf002.tif]

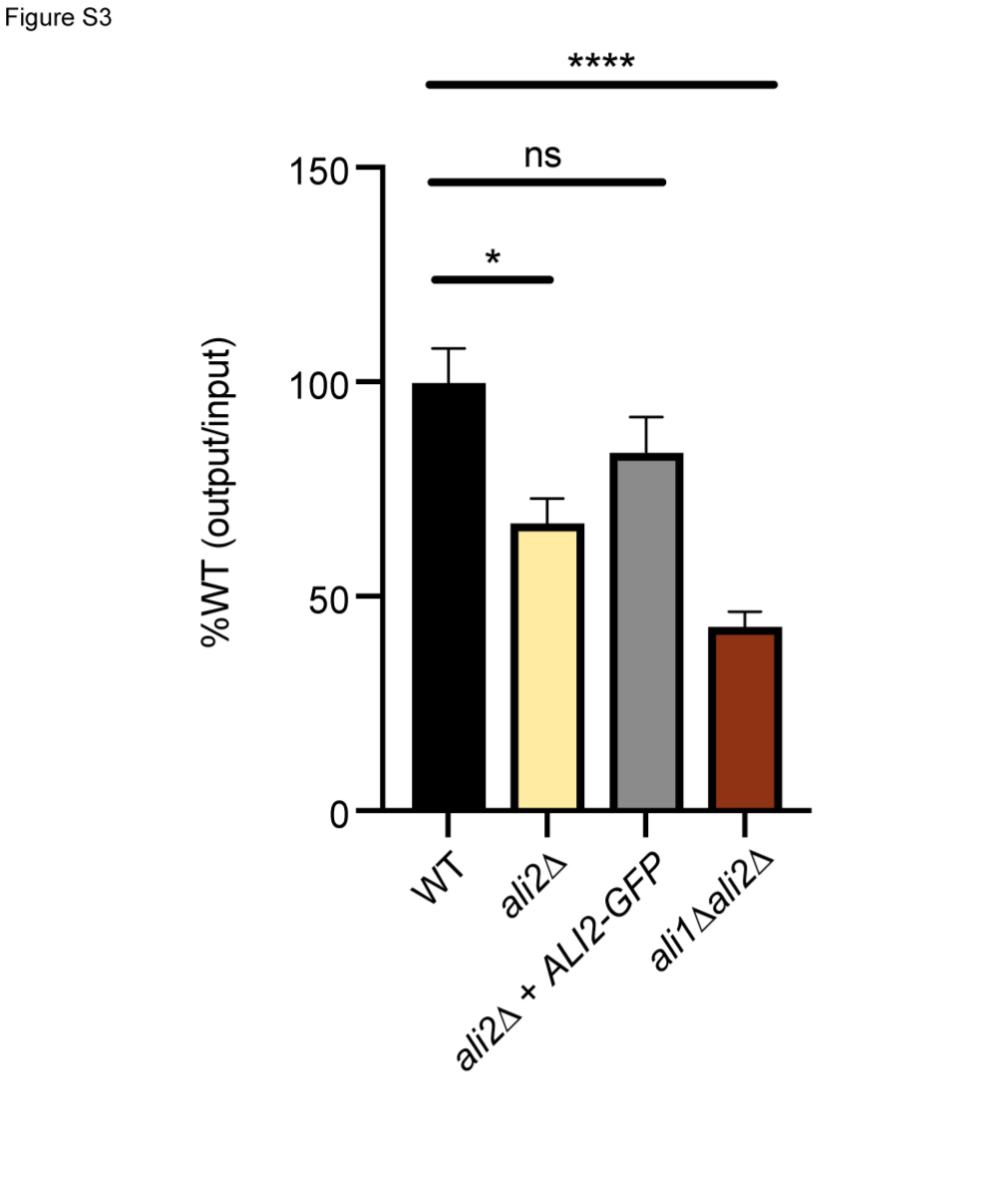

Supplement: FIG S3 [file mBio.02682-19-sf003.tif]

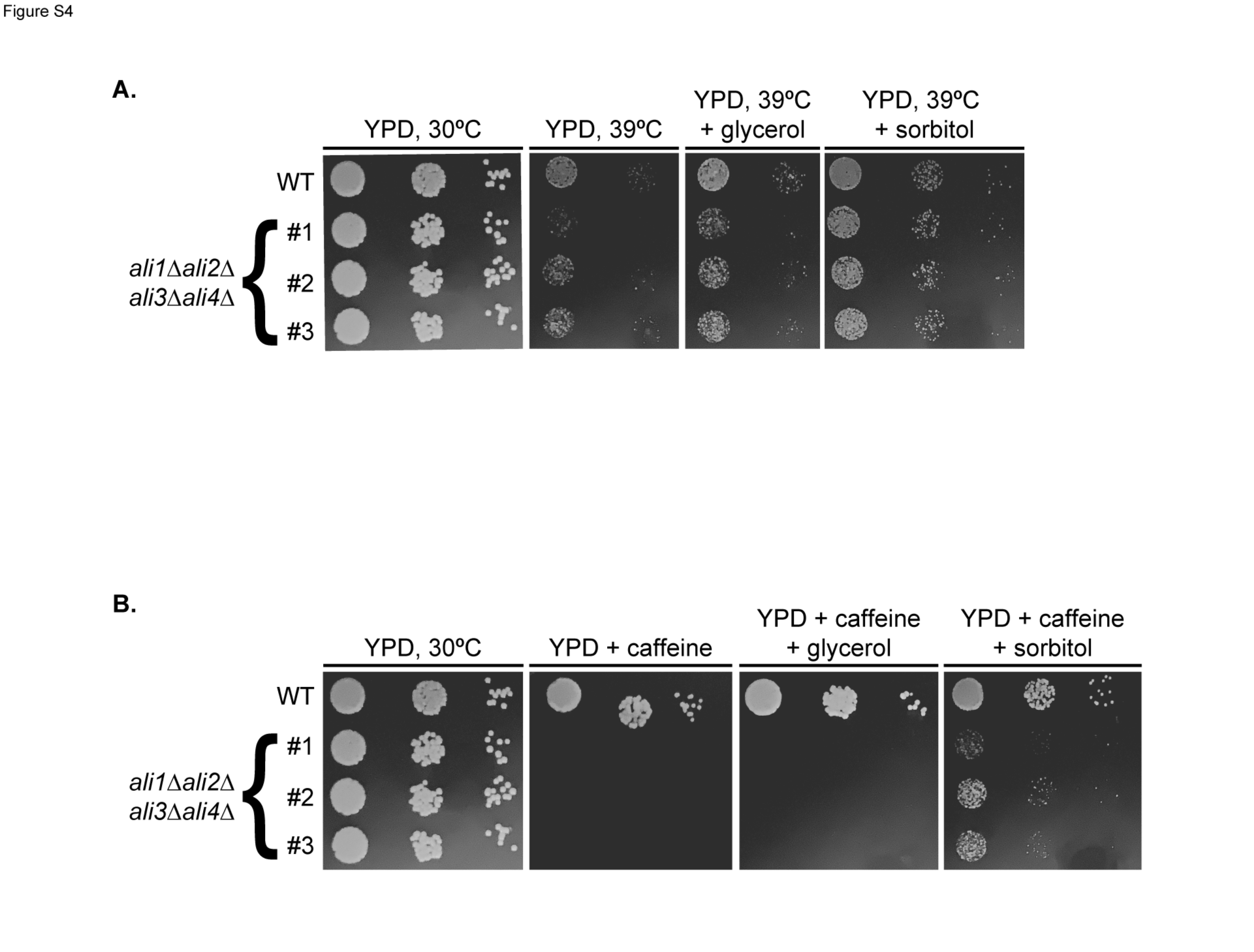

Supplement: FIG S4 [file mBio.02682-19-sf004.tif]
