# Supplementary material for: A Fungal Arrestin Protein Contributes to Cell Cycle Progression and Pathogenesis
Source: mBio. 2019 Nov 19;10(6):e02682-19. doi: 10.1128/mBio.02682-19 (PMC6867901; doi:10.1128/mBio.02682-19)
Supplement: FILE S1 — Supplemental materials and methods. Download FILE S1, DOCX file, 0.1 MB. [file mBio.02682-19-s0001.docx]

**File S1**. Supplementary materials and methods

**Molecular biology and strain construction**

To construct the loss-of-function mutants in this study – *ali1*Δ (KS120) and *ali2*Δ (KS96-2) – nourseothricin (NAT) or neomycin (NEO) dominant selectable markers were integrated into the genome by homologous recombination to replace the entire open reading frame (ORF) of the target gene (86). For the Cdc10 localization studies, the *ALI1* deletion construct was transformed into the Cdc10-mCherry (LK001) background to generate the Cdc10-mCherry + *ali1*Δ (CLT42) strain. The *ali3*Δ (CLT8) and *ali4*Δ (CLT9) loss-of-function mutants were obtained from the 2015 Madhani collection (87). All deletion strains were screened by NAT/NEO resistance and confirmed by PCR (both positive and negative confirmation PCRs demonstrating replacement of the respective wild-type [WT] locus with the deletion cassette), sequencing, and quantitative real time PCR.

Complemented strains – *ali1*Δ + *ALI1* (CLT6), *ali2*Δ + *ALI2-GFP* (CLT67)*, ali3*Δ + *ALI3* (CLT62)*, ali4*Δ + *ALI4* (CLT63) – were generated for each loss-of-function mutant by co-transformation with the respective WT ORF (with about 500 base pairs of 5’ UTR/promoter and 3’ UTR/terminator sequence) and the NAT or NEO dominant selectable markers. All complemented strains were screened by NAT/NEO resistance and confirmed by PCR (positive confirmation PCRs demonstrating the presence of the respective WT allele in the genome), quantitative real time PCR, and/or phenotypic rescue.

The Ali1-GFP (CLT7) strain was generated by transforming pCT3 into the *ali1*Δ (KS120) background. The pCT3 plasmid contains a C-terminally GFP-fused *ALI1* ORF under transcriptional control of the histone H3 promoter. About 500 base pairs of the *RRA1* 3’ UTR/terminator was included after the GFP sequence. The *ALI1* sequence was amplified from WT (H99) genomic DNA. The histone H3 sequence was amplified from pCN20. The GFP and *RRA1* 3’ UTR/terminator sequences were amplified from pKS85. These constructs were cloned in-frame into the PCR-linearized pUC19 backbone using In-Fusion cloning (Clontech). Transformants were screened by microscopy and phenotypic rescue, and confirmed by positive confirmation PCR, sequencing, quantitative real time PCR, and western blotting.

The Ali1-GFP + mCherry-Ras1 (CBN486) strain was constructed by genetic crossing between Ali1-GFP (CLT7) and mCherry-Ras1 (CBN327), in which *RAS1* is under transcriptional control of the galactose Gal7 promoter, on mating (MS) medium (88). Progeny were screened by colony morphology and microscopy, and subsequently confirmed by positive confirmation PCR. The arrestin null mutants were also generated by genetic crosses between a *MAT*α *ali1*Δ*ali2*Δ (KS97-2) mutant and a *MAT*a *ali3*Δ*ali4*Δ (CLT35) mutant, resulting in the isolation of multiple, independent arrestin null (CLT56, CLT57, and CLT58) mutants. Progeny were screened by NAT/NEO resistance, and confirmed by PCR (both positive and negative confirmation PCRs demonstrating replacement of the respective WT locus with the deletion cassette) and quantitative real time PCR.

**Proteomic experiment preparation and analysis**

Mass spectrometry analysis was performed on immunoprecipitations by the Duke Proteomics Core Facility, as described previously (26). The resin-bound immunoprecipitated proteins were treated with 1% RapiGest (Waters) at 50˚C for 10 minutes, reduced by the addition of 10 mM dithiothreitol and incubation at 32˚C for 35 minutes, and finally alkylated by the addition of 20 mM final concentration iodoacetamide and incubation at room temperature for 30 minutes. Subsequently, samples were trypsin digested on-resin overnight at 32˚C. Samples then underwent a 90-minute chromatographic separation using a nanoscale capillary reverse phase ultra-performance liquid chromatography system (Waters) in combination with a Q-Exactive Plus high-resolution accurate mass tandem mass spectrometer (Thermo Fisher) via a nanoelectrospray ionization source.

**Minimum inhibitory concentration (MIC) testing**

To measure strain susceptibilities to rapamycin and cerulenin, MIC testing was performed using species-specific modifications to standard CLSI testing methods for broth microdilution testing of antifungal susceptibility (91, 92). Briefly, the WT (H99), *ali1*Δ (KS120), and *ali1*Δ + *ALI1* (CLT6) strains were incubated to mid-logarithmic growth phase in YPD medium at 30°C. Cells were diluted to an OD_600_ of 0.25 in YPD medium (for rapamycin tests) or RPMI-1640 growth medium (for cerulenin tests). Rapamycin and cerulenin (Thermo-Fisher) were diluted in dimethyl sulfoxide (DMSO). Subsequently, 100-fold dilutions of the cell suspensions with serial dilutions of rapamycin at a dose range of 0.78-200 ng/mL were incubated at 30°C and 37°C. The 100-fold dilutions of the cell suspensions with serial dilutions of cerulenin at a dose range of 0.04-9.6 μg/mL were incubated at 30°C and 39°C. Growth was assessed by OD_600_ at 48 hours using a FLUOStar Optima plate reader. MIC_50_ values were calculated as described previously, by measuring relative growth of drug-treated cells compared to untreated cells, with MIC_50_ values corresponding to at least a 50% decrease in relative growth for the drug-treated cells.
